# Investigating the Effects of Intervention Strategies on Pneumonia and HIV/AIDS Coinfection Model

**DOI:** 10.1155/2023/5778209

**Published:** 2023-11-30

**Authors:** Shewafera Wondimagegnhu Teklu

**Affiliations:** Department of Mathematics, Natural Science, Debre Berhan University, Debre Berhan, Ethiopia

## Abstract

HIV/AIDS and pneumonia coinfection have imposed a major socioeconomic and health burden throughout the world, especially in the developing countries. In this study, we propose a compartmental epidemic model on the spreading dynamics of HIV/AIDS and pneumonia coinfection to investigate the impacts of protection and treatment intervention mechanisms on the coinfection spreading in the community. In the qualitative analysis of the model, we have performed the positivity and boundedness of the coinfection model solutions; the effective reproduction numbers using the next-generation operator approach; and both the disease-free and endemic equilibrium points' local and global stabilities using the Routh-Hurwiz and Castillo–Chavez stability criteria, respectively. We performed the sensitivity analysis of the model parameters using both the forward normalized sensitivity index criteria and numerical methods (simulation). Moreover, we carried out the numerical simulation for different scenarios to investigate the effect of model parameters on the associated reproduction number, the effect of model parameters on the model state variables, and the solution behavior and convergence to the equilibrium point(s) of the models. Finally, from the qualitative analysis and numerical simulation results, we observed that the disease-spreading rates, protection rates, and treatment rates are the most sensitive parameters, and we recommend for stakeholders to concentrate and exert their maximum effort to minimize the spreading rates by maximizing the protection and treatment rates.

## 1. Introduction

Infectious diseases investigated and verified in the laboratory or in the clinic are illnesses caused by pathogenic microorganisms, and pneumonia is an infectious disease caused by microorganisms like bacteria, virus, fungus, and parasites; HIV/AIDS is also an infectious disease caused by viruses [1–3].

Acquired immunodeficiency syndrome (AIDS) caused by human immunodeficiency virus (HIV), discovered in 1981, is one of the major deadly infectious diseases that has been spreading through countries in the world [1, 4–7]. Different literatures reported that HIV/AIDS has been the major health-affected infectious disease and affected more than seventy million individuals [1, 8, 9]. HIV attacks white blood cells and is spreading through sexual contact, sharing needle, and blood contact or by fluids containing the HIV virus and by vertical transmission from mother to child at birth [5, 6].

Pneumonia caused by various pathogenic microbial agents like virus, bacteria, fungi, and parasites is a major respiratory infectious disease identified as an inflammatory condition of the lungs [10–12]. Among the pathogenic microbial agents which have potential in causing pneumonia infection, bacteria especially *Streptococcus pneumoniae* have been reported as the leading cause [10–12]. The bacteria microbial agents enter the lungs, rapidly multiply its number, and settle in the air passage called alveoli of the human being lung; the lung will be filled with fluid and pus, which makes breathing difficult [10, 12]. Pneumonia is commonly a highly transmitted disease and a major cause of morbidity and mortality in both children and adults throughout nations in the world [13, 14].

Infectious disease studies using mathematical modelling approaches have been carried out by different researchers to tackle the basic epidemic problems and for making predictions of quantitative measures of different prevention and controlling strategies and their effectiveness; see literatures [1–13, 15–36]. Even though mathematical epidemiologists did not give attention like the common HIV/AIDS and TB coinfection [32, 34, 35] and other coinfections, the coinfection of HIV/AIDS and pneumonia in one host is a common phenomenon. Since pneumonia is one of the most common opportunistic infections for HIV/AIDS-infected individuals, some scholars have carried out few essential mathematical epidemiological research studies on the transmission dynamics of HIV/AIDS and pneumonia coinfection; see literatures [4, 5]. In this study, we have reviewed some epidemic mathematical modelling approach researches which are irrelevant to our proposed study and done by different scholars in the world. Huo et al. [1] presented a mathematical model approach study on a stage structure HIV/AIDS transmission dynamics of HIV/AIDS with treatment strategy. The finding of the study stated that the HIV/AIDS treatment strategy (ART) is the most effective strategy at the HIV asymptomatic stage of the HIV infection or before-AIDS stage to minimize its spreading in the community. Omondi et al. [9] presented a sex-structured community infection model and discuss male and female HIV infection trends with heterosexual activities. The finding of the study stated that the HIV/AIDS treatment (ART) strategy has a significant impact on controlling HIV/AIDS transmission in the community. Teklu [24] presented a mathematical modelling approach research on COVID-19 infection in the presence of prevention and control strategies. The results and findings of the study deduced that applying COVID-19 vaccination, other protection measures, home quarantine with treatment, and hospital quarantine with treatment simultaneously is the most effective strategy to minimize the COVID-19 spread in the community. Teklu and Mekonnen [4] analyzed HIV/AIDS and pneumonia coinfection model with treatment at each infection stage. From the results of the model analysis, they deduced that applying treatment mechanisms for both the single infections and coinfection individuals is the most effective strategy to minimize the coinfection disease-spreading dynamics. Teklu and Rao [5] proposed and investigated a compartmental model on the coexistence of HIV/AIDS and pneumonia with pneumonia vaccination, treatments of pneumonia, and HIV/AIDS infection control measures. The finding of the model analysis stated that to minimize the coinfection disease spread in the community, controlling pneumonia infection using vaccination and treatment is more effective than treatment of HIV/AIDS only infection.

The main purpose of this study is to investigate the impacts of pneumonia protection, pneumonia treatment, and HIV protection by using condom and HIV treatment (ART) intervention strategies simultaneously on the transmission dynamics of HIV/AIDS and pneumonia coinfection in the community. Even though researchers [4, 5] invested much effort in studying HIV/AIDS and pneumonia coinfection, they did not consider pneumonia protection, pneumonia vaccination, pneumonia treatment, HIV protection by using condom, and HIV treatment as prevention and control strategies simultaneously in their proposed coinfection model formulation and analysis. And also, the main contributions of this study are as follows: the health stakeholders can use the findings of this modified research study to tackle the HIV/AIDS and pneumonia coinfection in the community; potential young researchers can develop their epidemiological modelling knowledge and skills; and potential senior researchers can modify the study by incorporating different modelling and intervention aspects. Based on the findings of the above-reviewed literatures, we have realized the gaps and are highly motivated to tackle the problem by modifying the research study [5]. The remaining part of this study is structured in the following sequence: the model is formulated in Section 2 and is analyzed in Section 3; sensitivity analysis, numerical simulation, and conclusions of the study are carried out in Sections 4 and 5, respectively.

## 2. Model Description and Formulation

Motivated by various scholars' mathematical modelling researches in real-world situations, we proposed a coinfection integer order model on HIV/AIDS and pneumonia spreading dynamics. To describe and formulate the proposed coinfection model, we divide the total human population considered in this study at a time *t* and represented by *N*(*t*) into nine mutually distinct classifications as follows: the number of people who are susceptible to either HIV/AIDS or pneumonia infection represented by *S*(*t*), the number of people who are protected against pneumonia infection represented by *P*_*P*_(*t*), the number of people who are protected against HIV infection by using condom is represented by *H*_*P*_(*t*), the number of people who are infected with pneumonia is only represented by *P*_*I*_(*t*), the number of people who are infected with HIV is only represented by *H*_*I*_(*t*), the number of people who are AIDS patients represented by *H*_*A*_(*t*), the number of people who are coinfected with HIV/AIDS and pneumonia is represented by *C*(*t*), the number of people who are treated from HIV/AIDS is represented by *T*_*HA*_(*t*), the number of people who are infected with pneumonia is represented by *P*_*T*_(*t*); and the total number of individuals who are considered in this study is represented by

(1) $$\begin{array}{c} N\left(t\right)=S\left(t\right)+P_{P}\;\left(t\right)+H_{P}\left(t\right)+H_{I}\left(t\right)+H_{A}\left(\text{t}\right)+P_{I}\left(t\right)+C\left(t\right)+P_{T}\left(t\right)+T_{\text{HA}}\left(t\right). \end{array}$$

The force of infection where the susceptible people acquire HIV/AIDS is defined by

(2) $$\begin{array}{c} \lambda_{H}\left(t\right)=\frac{\beta_{1}}{N}\left(H_{I}\left(t\right)+\alpha H_{A}\left(t\right)+\vartheta C\left(t\right)\right). \end{array}$$

where 1 ≤ *σ* < ∞ and 1 ≤ *ϑ* < ∞ are the modification parameters which increase infectivity of individuals and *β*_1_ is the HIV/AIDS spreading rate.

The force of infection where the susceptible people acquire pneumonia is defined by

(3) $$\begin{array}{c} \lambda_{P}\left(\text{t}\right)=\frac{\beta_{2}}{N}\left(P_{I}\left(t\right)+\omega C\left(t\right)\right). \end{array}$$

where 1 ≤ *ω* < ∞ is the modification parameter which increases infectivity and *β*_2_ is the pneumonia spreading rate.

To formulate the proposed coinfection model of HIV/AIDS and pneumonia, let us assume the following: *p*_1_, *p*_2_, and (1 − *p*_1_ − *p*_2_) be portions of the total recruited people Γ who are entering to the pneumonia-protected class *P*_*P*_(*t*), to the HIV protected class *H*_*P*_(*t*), and to the susceptible class *S*(*t*), respectively; pneumonia recovery by treatment is not permanent, human population is homogeneous and is not constant, there is no HIV transmission from HIV-treated people and no HIV vertical transmission, and there is no simultaneous HIV and pneumonia dual-infection transmission.

**Figure 1:**
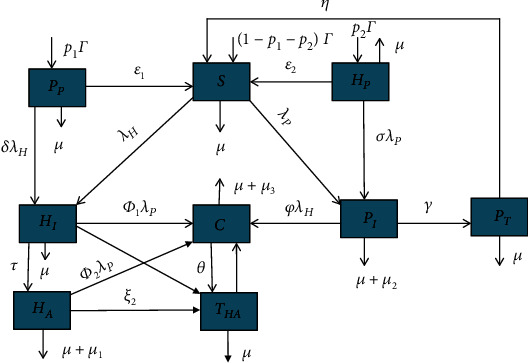
The flow chart of the HIV/AIDS and pneumonia codynamics spreading dynamics with forces of infections *λ*_*H*_(*t*) and *λ*_*P*_(*t*) given in (2) and (3), respectively.

Based on Tables 1 and 2 and the model descriptions and assumptions given above, the flow chart for the spreading dynamics of HIV/AIDS and pneumonia coinfection is illustrated in Figure 1.

Based on Figure 1, we derive the system of nonlinear differential equations of the coinfection model as follows:

(4) $$\begin{array}{c} \frac{dS}{dt}=\left(1-p_{1}-p_{2}\right)\Gamma +\varepsilon_{1}P_{P}+\varepsilon_{2}H_{P}+\eta {\text{P}}_{T}-\left(\lambda_{H}+\lambda_{P}+\mu \right)S,\\ \frac{dP_{P}}{dt}=p_{1}\Gamma -\left(\delta \lambda_{H}+\varepsilon_{1}+\mu \right)P_{P},\\ \frac{dH_{P}}{dt}=p_{2}\Gamma -\left(\varepsilon_{2}+\mu +\sigma \lambda_{P}\right)H_{P},\\ \frac{dH_{I}}{dt}=\lambda_{H}S+\delta \lambda_{H}P_{P}-\left(\mu +\tau +\xi_{1}+\phi_{1}\lambda_{P}\right)H_{I},\\ \frac{dH_{A}}{dt}=\tau H_{I}-\left(\mu +\mu_{1}+\xi_{2}+\phi_{2}\lambda_{P}\right)H_{A},\\ \frac{\text{d}{\text{P}}_{\text{I}}}{\text{dt}}=\lambda_{\text{P}}\text{S}+\sigma \lambda_{\text{P}}{\text{H}}_{\text{P}}-\left(\mu +\mu_{2}+\gamma +\varphi \lambda_{\text{H}}\right){\text{P}}_{\text{I}},\\ \frac{dC}{dt}=\varphi \lambda_{H}P_{I}+\phi_{1}\lambda_{P}H_{I}+\phi_{2}\lambda_{P}H_{A}+\rho \lambda_{P}T_{HA}-\left(\mu +\mu_{3}+\theta \right)C,\\ \frac{dP_{T}}{dt}=\gamma P_{I}-\left(\mu +\eta \right)P_{T},\\ \frac{\text{d}{\text{T}}_{\text{HA}}}{\text{dt}}=\xi_{1}{\text{H}}_{\text{I}}+\xi_{2}{\text{H}}_{\text{A}}+\theta \text{C}-\left(\mu +\rho \lambda_{\text{P}}\right){\text{T}}_{\text{HA}}, \end{array}$$

with initial data,

(5) $$\begin{array}{c} \;S\left(0\right)>0,\\ P_{P}\left(0\right)\geq 0,\\ H_{P}\left(0\right)\geq 0,\\ H_{I}\left(0\right)\geq 0,\\ H_{A}\left(0\right)\geq 0,\\ H_{I}\left(0\right)\geq 0,\\ C\left(0\right)\geq 0,\\ \;P_{T}\left(0\right)\geq 0,\\ \;T_{HA}\left(0\right)\geq 0. \end{array}$$

Adding all the differential equations in the system, (4) gives

(6) $$\begin{array}{c} \frac{dN}{dt}=\Delta -\mu N-\left(\mu_{1}H_{A}+\mu_{2}P_{I}+\mu_{3}C\right). \end{array}$$

## 3. Qualitative Analysis of the Model (4)

### 3.1. Nonnegativity and Boundedness of the Model Solutions

Since the proposed model (4) deals with human beings, we need to investigate that each of the model solution variables is nonnegative and bounded in the region.

(7) $$\begin{array}{c} \Omega =\left\{\left(S,P_{P},H_{P},H_{I},H_{A},P_{I},C,P_{T},T_{HA}\right)\in {\mathbb{R}}_{+}^{9},N\leq \frac{\Gamma }{\mu }\right\}. \end{array}$$

**Theorem 1 .**

Nonnegativity.

Depending on the initial data given in equation (5), each of the model solutions *S*(*t*), *P*_*P*_(*t*), *H*_*P*_(*t*),  *H*_*I*_(*t*), *H*_*A*_(*t*), *P*_*I*_(*t*),*C*(*t*), *P*_*T*_(*t*), and  *T*_*HA*_(*t*) of the system (4) is nonnegative for *t* > 0.

**Proof**

Let the initial data be  *S*(0) > 0, *P*_*P*_(0) > 0, *H*_*P*_(0) > 0, *H*_*I*_(0) > 0, *H*_*A*_(0) > 0, *P*_*I*_(0) > 0,  *C*(0) > 0,  *P*_*T*_(0) > 0, and *T*_*HA*_(0) > 0. Then, *t* > 0, we need to prove that all the model solutions *S* (*t*) > 0, *P*_*P*_(*t*) > 0, *H*_*P*_(*t*) > 0,  *H*_*I*_(*t*) > 0,  *H*_*A*_(*t*) > 0, *P*_*I*_(*t*) > 0,  *C*(*t*) > 0,  *P*_*T*_(*t*) > 0, and *T*_*HA*_(*t*) > 0.

Now, let us define the following set: *τ* = sup{*t* > 0 : *S* (t) > 0, *P*_*P*_(*t*) > 0, *H*_*P*_(*t*) > 0, *H*_*I*_(*t*) > 0, *H*_*A*_(*t*) *P*_*I*_(*t*) > 0, *C*(*t*) > 0, *P*_*T*_(*t*) > 0 and *T*_*HA*_(*t*) > 0}. Because the model state variables *S*(*t*), *P*_*P*_(*t*), *H*_*P*_(*t*), *H*_*I*_(*t*), *H*_*A*_(*t*) *P*_*I*_(*t*), *C*(*t*), *R*_*T*_(*t*) and *T*_*HA*_(*t*) are continuous, we deduce that *τ* > 0.

If *τ* = ∞, then nonnegativity holds. But, if 0 < *τ* < ∞, *S*(*τ*) = 0 or *P*_*P*_ (*τ*) = 0 or *H*_*P*_ (*τ*) = 0 or *H*_*I*_(*τ*) = 0, *H*_*A*_(*τ*) = 0 or  *P*_*I*_( *τ* ) = 0 or *C*(*τ*) = 0  or  *P*_*T*_(*τ*) = 0 or *T*_*HA*_(0) = 0.

Rearranging the first equation of the model (4) gives us

(8) $$\begin{array}{c} \frac{dS}{dt}+\left(\lambda_{H}+\lambda_{P}+\mu \right)S=\left(1-p_{1}-p_{2}\right)\Gamma +\varepsilon_{1}P_{P}+\varepsilon_{2}H_{P}+\eta P_{T}. \end{array}$$

We apply the method of integrating factors, and after some computations, we determined the result *S*(*τ*) = *M*_1_*S*(0) + *M*_1_∫_0_^*τ*^exp^∫(*λ*_*H*_ + *λ*_*P*_ + *μ*))*dt*^((1 − *p*_1_ − *p*_2_)Γ + *ε*_1_*P*_*P*_(t) + *ε*_2_*H*_*P*_(t) + *η*P_*T*_(t))*dt* > 0, where *M*_1_ = exp^−(*μτ* + ∫_0_^*τ*^(*λ*_*H*_(*w*) + *λ*_*P*_(*w*))^ > 0, *S*(0) > 0, *P*_*P*_(t) > 0, *H*_*P*_(t) > 0, P_*T*_(t) > 0, and by the meaning of *τ*, *S*(*τ*) > 0, hence *S*(*τ*) ≠ 0.

Similarly, rearranging the second equation of the system (4) gives us (*dP*_*P*_/*dt*) + (*δλ*_*H*_ + *ε*_1_ + *μ*)*P*_*P*_ = *ε*_1_Γ , and we have got *P*_*P*_(*τ*) = *M*_1_*P*_*P*_(0) + *M*_1_∫_0_^*τ*^exp^∫(*ε*_1_ + *μ* + *δλ*_*H*_(*t*))*dt*^*p*_1_Δ*dt* > 0, where *M*_1_ = exp^−(*ε*_1_*τ* + *μτ* + ∫_0_^*τ*^(*δλ*_*H*_(*w*))^ > 0, *P*_*P*_(0) > 0, by definition of *τ*, *P*_*P*_(*τ*) > 0, hence *P*_*P*_(*τ*) ≠ 0.

Similarly, one can determine the results; *H*_*P*_(*τ*) > 0 hence *H*_*P*_(*τ*) ≠ 0, *H*_*I*_(*τ*) > 0 hence *H*_*I*_(*τ*) ≠ 0, *P*_*I*_(*τ*) > 0 hence *P*_*I*_(*τ*) ≠ 0, *C*(*τ*) > 0 hence *C*(*τ*) ≠ 0, *P*_*T*_(*τ*) > 0 hence *P*_*T*_(*τ*) ≠ 0, and *T*_*HA*_(*τ*) > 0 hence *T*_*HA*_(*τ*) ≠ 0. Thus, by definition of *τ* given above, *τ* = ∞, and hence, each of the model (3) solutions is nonnegative.

**Theorem 2 Boundedness.**

Each of the model solutions given in the region *Ω* (7) is bounded in  ℝ_+_^9^.

**Proof**

In the absence of infections, the sum of all the differential equations given in (6), and by the nonnegativity condition in Theorem 1, we have (*dN*/*dt*) ≤ Γ − *μN*. Based on the concept of the standard comparison theorem, we determined the result ∫(*dN*/Γ − *μN*) ≤ ∫*dt*, and integrating both sides gives us the result −(1/*μ*)ln(Γ − *μN*) ≤ *t* + *c*, where *c* is some constant, and after some computations, we have the result

0 ≤ *N* (*t*) ≤ (Γ/*μ*). Therefore, the model (4) solutions with positive initial data given in (5) are bounded.

### 3.2. Qualitative Analysis HIV/AIDS Infection Submodel

Now, make the state variables corresponding to pneumonia-only infection of the coinfection model (4) as *P*_*P*_ = *P*_*I*_ = *C* = *P*_*T*_ = 0, we have the HIV/AIDS infection submodel given by

(9) $$\begin{array}{c} \frac{dS}{dt}=\left(1-p_{2}\right)\;\Gamma +\varepsilon_{2}H_{P}-\left(\lambda_{H}+\mu \right)S,\\ \frac{dH_{P}}{dt}=p_{2}\Gamma -\left(\varepsilon_{2}+\mu \right)H_{P},\\ \frac{dH_{I}}{dt}=\lambda_{H}S-\left(\mu +\xi_{1}+\tau \right)H_{I},\\ \frac{\text{d}{\text{H}}_{\text{A}}}{\text{dt}}=\tau {\text{H}}_{\text{I}}-\left(\mu +\mu_{1}+\xi_{2}\right){\text{H}}_{\text{A}},\\ \frac{dT_{HA}}{dt}=\xi_{1}H_{I}+\xi_{1}H_{A}-\mu T_{HA}, \end{array}$$

where *λ*_*H*_(*t*) = (*β*_1_/*N*_1_)(*H*_*I*_(*t*) + *αH*_*A*_(*t*)) and *N*_1_ = *S* + *H*_*P*_ + *H*_*I*_ + *H*_*A*_ + *T*_*HA*_.

#### 3.2.1. Local Stability of Disease-Free Equilibrium Point

The HIV/AIDS submodel (8) disease-free equilibrium (DFE) is computed by making each equation of the dynamical system (8) equal to zero where there are no infections and treated groups. Therefore, the submodel (8) DFE is given by  *E*_*H*_^0^ = (S^0^, *H*_*P*_^0^, *H*_*I*_^0^, *H*_*A*_^0^, *T*_*HA*_^0^) = ((Γ/*μ*)(*ε*_2_ + *μ*(1 − *p*_2_)/*ε*_2_ + *μ*), (*p*_2_Γ/*ε*_2_ + *μ*), 0, 0, 0).

Using the same method stated in [22] on the HIV/AIDS submodel (8), we have computed the matrices *F* and *V* by

(10) $$\begin{array}{c} F=\left(\begin{array}{ccc} \frac{\beta_{1}}{N_{1}^{0}}{\text{S}}^{0} & \frac{\beta_{1}}{N_{1}^{0}}\alpha {\text{S}}^{0} & 0\\ 0 & 0 & 0\\ 0 & 0 & 0 \end{array}\right)=\left(\begin{array}{ccc} \frac{\beta_{1}\varepsilon_{2}+\beta_{1}\mu \left(1-p_{2}\right)}{\varepsilon_{2}+\mu } & \frac{\beta_{1}\varepsilon_{2}\alpha +\beta_{1}\mu \alpha \left(1-p_{2}\right)}{\varepsilon_{2}+\mu } & 0\\ 0 & 0 & 0\\ 0 & 0 & 0 \end{array}\right),\\ V=\left(\begin{array}{ccc} \mu +\xi_{1}+\tau & 0 & 0\\ -\tau & \mu +\mu_{1}+\xi_{2} & 0\\ -\xi_{1} & -\xi_{2} & \mu \end{array}\right). \end{array}$$

The HIV/AIDS submodel (8) basic reproduction number is the largest eigenvalue in magnitude of the next generation matrix *FV*^−1^ and is computed as

(11) $$\begin{array}{c} R_{H}^{0}=\frac{\beta_{1}\left(\mu \left(1-p_{2}\right)+\varepsilon_{2}\right)}{\left(\mu +\xi_{1}+\tau \right)\left(\mu +\varepsilon_{2}\right)}+\frac{\beta_{1}\mu \alpha \left(1-p_{2}\right)+\left.\beta_{1}\varepsilon_{2}\alpha \right)}{\left(\mu +\mu_{1}+\xi_{2}\right)\left(\mu +\varepsilon_{2}\right)} \end{array}$$

The threshold quantity *ℛ*_*H*_^0^ (basic reproduction number) of the HIV/AIDs submodel (8) is the expected number of secondary HIV infections produced by single infected human during its entire period of infectiousness throughout the whole susceptible community, and the HIV/AIDS submodel disease-free equilibrium point *E*_*H*_^0^ = (S^0^, *H*_*P*_^0^, *H*_*I*_^0^, *H*_*A*_^0^, *T*_*HA*_^0^) = (Γ/*μ*((*ε*_2_ + *μ*(1 − *p*_2_)/*ε*_2_ + *μ*)), (*p*_2_Γ/*ε*_2_ + *μ*), 0, 0, 0) has a local asymptotic stability whenever *ℛ*_*H*_^0^ < 1, and unstable whenever *ℛ*_*H*_^0^ > 1.

#### 3.2.2. Endemic Equilibrium Point (*S*) Existence and Uniqueness

In this subsection setting, the right-hand side of the HIV/AIDS-only dynamical system given in equation (9) is equal to zero, and after a number of steps of computations, we have determined the endemic equilibrium point(s) given by

(12) $$\begin{array}{c} S^{*}=\frac{D_{1}}{D_{2}\left(\mu +\lambda_{H}^{*}\right)},H_{P}^{*}=\frac{p_{2}\Gamma }{D_{2}},H_{I}^{*}=\frac{D_{1}\lambda_{H}^{*}}{D_{2}D_{3}\left(\mu +\lambda_{H}^{*}\right)},H_{A}^{*}=\frac{D_{1}\tau \lambda_{H}^{*}}{D_{2}D_{3}D_{4}\left(\mu +\lambda_{H}^{*}\right)},\text{and }T_{\text{HA}}^{*}=\frac{D_{1}D_{4}\xi_{1}\lambda_{H}^{*}+D_{1}\tau \xi_{2}\lambda_{H}^{*}}{D_{2}D_{3}\mu \left(\mu +\lambda_{H}^{*}\right)}, \end{array}$$

where  *D*_1_ = *ε*_2_Γ + *μ*Γ(1 − *p*_2_),  *D*_2_ = (*ε*_2_ + *μ*),  *D*_3_ = (*μ* + *ξ*_1_ + *τ*),  *D*_4_ = (*μ* + *μ*_1_ + *ξ*_2_).

Now, substitute *H*_*I*_^∗^ and *H*_*A*_^∗^ in the HIV/AIDS force of infection given by *λ*_*H*_^∗^ = (*β*_1_/*N*_1_^∗^)(*H*_*I*_^∗^ + *αH*_*A*_^∗^), and computing for *λ*_*H*_^∗^, we have determined that

(13) $$\begin{array}{c} \lambda_{H}^{*}=\frac{=-D_{3}D_{4}\mu }{D_{1}D_{2}\tau \mu \Gamma \left(1-\left(\beta_{1}\left(\mu \left(1-p_{2}\right)+\varepsilon_{2}\right)/\left(\mu +\xi_{1}+\tau \right)\left(\mu +\varepsilon_{2}\right)\right)-\left(\beta_{1}\mu \alpha \left(1-p_{2}\right)+\left.\beta_{1}\varepsilon_{2}\alpha \right)/\left(\mu +\mu_{1}+\xi_{2}\right)\left(\mu +\varepsilon_{2}\right)\right)\right)}=\frac{-D_{3}D_{4}\mu }{D_{1}D_{2}\tau \mu \Gamma \left(1-R_{H}^{0}\right)}>0 \end{array}$$

if and only if *ℛ*_*H*_^0^ > 1.

Thus, based on the final result *λ*_*H*_^∗^ > 0, there is a unique positive endemic equilibrium for the HIV/AIDS submodel given in equation (9) if and only if *ℛ*_*H*_^0^ > 1.

**Lemma 1 .**

The HIV/AIDS monoinfection model given in equation (9) has a unique endemic equilibrium solution if and only if *ℛ*_*H*_^0^ > 1.

#### 3.2.3. DFE Global Asymptotic Stability

**Lemma 2 (The Castillo-Chavez et al. criteria stated in [23]).**

If the HIV/AIDS submodel can be written as

(14) $$\begin{array}{c} \frac{\text{dX}}{\text{dt}}=\text{I}\left(\text{X},\text{V}\right),\\ \frac{\text{dV}}{\text{dt}}=\text{J}\left(\text{X},\text{V}\right),\text{H}\left({\text{X}}^{0},0\right)=0, \end{array}$$

where *X* ∈ ℝ^*m*^ be the components of noninfected individuals and *V* ∈ ℝ^*n*^ be the components of infected individuals including treated class, and *E*_*H*_^0^ = (*X*^0^, 0) denotes the disease-free equilibrium point of the dynamical system (7).

Assume (i) for (*dX*/*dt*) = *I*(*X*^0^, 0),  *Y*^0^ is globally asymptotically stable (GAS). (ii) $J\left(X,V\right)=BV-\overset{ˇ}{J}\left(X,V\right)$,$\;\overset{ˇ}{J}\left(X,V\right)\geq 0$ for (*X*, *V*) ∈ *Ω*_1_ where *B* = *D*_*V*_*J*(*X*^0^, 0) is an M-matrix, i.e., the off-diagonal elements of *B* are nonnegative, and *Ω*_1_ is the region in which the system makes biological sense. Then, the fixed point *E*_*H*_^0^ = (*X*^0^, 0) is globally asymptotically stable equilibrium point of the system (8) whenever *ℛ*_*H*_^0^ < 1.

**Lemma 3 .**

The HIV/AIDS submodel disease-free equilibrium point *E*_*H*_^0^ = (Γ/*μ*(*ε*_2_ + *μ*(1 − *p*_2_)/*ε*_2_ + *μ*), (*p*_2_Γ/*ε*_2_ + *μ*), 0, 0, 0) is globally asymptotically stable if *ℛ*_*H*_^0^ < 1 and the two sufficient conditions given in Lemma 4 are satisfied.

**Proof**

To prove Lemma 5, let us apply Lemma 4 on the HIV/AIDS infection submodel (8), and we have determined the following matrices:

(15) $$\begin{array}{c} \frac{dX}{dt}=I\left(X,V\right)=\left[\begin{array}{c} \left(1-p_{2}\right)\Gamma +\varepsilon_{2}H_{P}-\left(\lambda_{H}+\mu \right)S\\ \varepsilon_{2}\Gamma -\left(\varepsilon_{2}+\mu \right)H_{P} \end{array}\right],\\ \frac{dV}{dt}=J\left(X,V\right)=\left[\begin{array}{c} \lambda_{H}S-\left(\mu +\xi_{1}+\tau \right)H_{I}\\ \tau H_{I}-\left(\mu +\mu_{1}+\xi_{2}\right)H_{A}\\ \xi_{1}H_{I}+\xi_{1}H_{A}-\mu T_{HA} \end{array}\right],\\ I\left(X^{0},0\right)=\left[\begin{array}{c} \left(1-p_{2}\right)\Gamma +\varepsilon_{2}H_{P}^{0}-\mu S^{0}\\ \varepsilon_{2}\Gamma -\left(\varepsilon_{2}+\mu \right)H_{P}^{0} \end{array}\right], \end{array}$$

where

(16) $$\begin{array}{c} X^{0}=\left(S^{0},H_{P}^{0}\right)=\left(\frac{\Gamma }{\mu }\left(\frac{\varepsilon_{2}+\mu \left(1-p_{2}\right)}{\varepsilon_{2}+\mu }\right),\frac{p_{2}\Gamma }{\varepsilon_{2}+\mu }\right) \end{array}$$

is globally stable which satisfies condition (i) of Lemma 4 and

(17) $$\begin{array}{c} B=D_{V}J\left(X^{0},0\right)=\left[\begin{array}{ccc} \frac{\beta_{1}S^{0}}{N_{1}^{0}}-\left(\mu +\xi_{1}+\tau \right) & \frac{\beta_{1}{\alpha S}^{0}}{N_{1}^{0}} & 0\\ \tau & -\left(\mu +\mu_{1}+\xi_{2}\right) & 0\\ \xi_{1} & \xi_{2} & -\mu \end{array}\right]. \end{array}$$

After a number of steps of computations, we have determined the result given by

(18) $$\begin{array}{c} \overset{ˇ}{J}\left(X,V\right)=\left[\begin{array}{c} {\overset{ˇ}{J}}_{1}\left(X,V\right)\\ {\overset{ˇ}{J}}_{2}\left(X,V\right)\\ {\overset{ˇ}{j}}_{3}\left(X,V\right) \end{array}\right]=\left[\begin{array}{c} \frac{\beta_{1}S^{0}}{N_{1}^{0}}H_{I}+\frac{\beta_{1}{\alpha S}^{0}}{N_{1}^{0}}H_{A}-\frac{\beta_{1}}{N_{1}}H_{I}S-\frac{\beta_{1}\alpha }{N_{1}}H_{A}S\\ 0\\ 0 \end{array}\right]=\left[\begin{array}{c} \beta_{1}H_{I}\left(\frac{S^{0}}{N_{1}^{0}}-\frac{S}{N_{1}}\right)+\beta_{1}\alpha H_{A}\left(\frac{S^{0}}{N_{1}^{0}}-\frac{S}{N_{1}}\right)\\ 0\\ 0 \end{array}\right]. \end{array}$$

From the definitions of state variables and total population, we can justify the inequality *S* ≤ *N*_1_ that implies *S*/*N*_1_ ≤ *S*^0^/*N*_1_^0^ and hence ${\overset{ˇ}{J}}_{1}\left(X,V\right)\geq 0$, which satisfies criteria (ii) of Lemma 4; thus, the HIV/AIDS submodel (8) disease-free equilibrium point *E*_*H*_^0^ = (S^0^, *H*_*P*_^0^, *H*_*I*_^0^, *H*_*A*_^0^, *T*_*HA*_^0^) = (Γ/*μ*(*ε*_2_ + *μ*(1 − *p*_2_)/*ε*_2_ + *μ*), (*p*_2_Γ/*ε*_2_ + *μ*), 0, 0, 0) is globally asymptotically stable if *ℛ*_*H*_^0^ < 1.

Epidemiologically, it means whenever *ℛ*_*H*_^0^ < 1, the HIV/AIDS-only disease dies out while the total population increases.

### 3.3. Qualitative Analysis of Pneumonia Infection Submodel

Now, making all the state variables corresponding to HIV/AIDS infection in the full model (4) as *H*_*P*_ = *H*_*I*_ = *H*_*A*_ = *C* = *T*_*HA*_ = 0, we have the pneumonia submodel given by

(19) $$\begin{array}{c} \frac{dS}{dt}=\left(1-p_{1}\right)\Gamma +\varepsilon_{1}P_{P}+\eta {\text{P}}_{R}-\left(\lambda_{P}+\mu \right)S,\\ \frac{dP_{P}}{dt}=p_{1}\Gamma -\left(\varepsilon_{1}+\mu \right)P_{P},\\ \frac{dP_{I}}{dt}=\lambda_{P}S-\left(\mu +\mu_{2}+\gamma \right)P_{I},\\ \frac{dP_{T}}{dt}=\gamma P_{I}-\left(\mu +\eta \right)P_{T}, \end{array}$$

with force of infection illustrated by

(20) $$\begin{array}{c} \lambda_{P}=\frac{\beta_{2}}{N_{2}}P_{I}\left(t\right), \end{array}$$

and with initial data *S*(0) > 0, *P*_*P*_(0) ≥ 0, *P*_*I*_(0) ≥ 0, *P*_*T*_(0) ≥ 0, total number of human beings involved is given by *N*_2_(*t*) = *S*(*t*) + *P*_*P*_(*t*) + *P*_*I*_(*t*) + *P*_*T*_(*t*).

#### 3.3.1. Local Stability of Disease-Free Equilibrium Point

The pneumonia submodel (19) disease-free equilibrium point is computed by making the model equations equal to zero, where *P*_*I*_ = *P*_*T*_ = 0. Thus, the pneumonia submodel disease-free equilibrium point is given by *E*_*P*_^0^ = (S^0^, *P*_*P*_^0^, *P*_*I*_^0^, P_T_^0^) = (Γ/*μ*(*ε*_1_ + *μ*(1 − *p*_1_)/*ε*_1_ + *μ*), *p*_1_Γ/*ε*_1_ + *μ*, 0, 0).

The pneumonia submodel (19) basic reproduction number is the estimated number of new pneumonia-infected individuals produced by one infectious individual in a community. Similarly, using the same criteria stated in [22], we computed the pneumonia reproduction number given by *ℛ*_*P*_^0^ = *β*_2_(*μ*(1 − *p*_1_) + *ε*_1_)/(*μ* + *μ*_2_ + *γ*)(*μ* + *ε*_1_).

The pneumonia submodel (19) basic reproduction number is defined as the estimated number of secondary infected individuals produced by single infectious individual during its entire period of infectiousness throughout the whole susceptible population, and using the same criteria, the disease-free equilibrium point given by *E*_*P*_^0^ = (S^0^, *P*_*P*_^0^, *P*_*I*_^0^, P_T_^0^) = (Γ/*μ*(*ε*_1_ + *μ*(1 − *p*_1_)/*ε*_1_ + *μ*), (*p*_1_Γ/*ε*_1_ + *μ*), 0, 0) is locally asymptotically stable whenever *ℛ*_*P*_^0^ < 1 and unstable whenever *ℛ*_*P*_^0^ > 1.

#### 3.3.2. Existence and Uniqueness of Endemic Equilibrium Point

The endemic equilibrium points of the pneumonia submodel given in equation (19) are computed by making the right-hand side of the system as zero, and after some computations, we have determined that

(21) $$\begin{array}{c} S^{*}=\frac{\left(1-p_{1}\right)\Gamma K_{1}K_{2}K_{3}+\varepsilon_{1}p_{1}\Gamma K_{2}K_{3}}{K_{1}K_{2}K_{3}\left(\lambda_{P}^{*}+\mu \right)-K_{1}\eta \gamma \lambda_{P}^{*}},\\ P_{P}^{*}=\frac{p_{1}\Gamma }{K_{1}},P_{I}^{*}=\frac{\left(1-p_{1}\right)\Gamma K_{1}K_{2}K_{3}\lambda_{P}^{*}+\varepsilon_{1}p_{1}\Gamma K_{2}K_{3}\lambda_{P}^{*}}{K_{1}{K_{2}}^{2}K_{3}\left(\lambda_{P}^{*}+\mu \right)-K_{1}K_{2}\eta \gamma \lambda_{P}^{*}},\\ P_{T}^{*}=\frac{\left(1-p_{1}\right)\Gamma K_{1}K_{2}K_{3}\gamma \lambda_{P}^{*}+\varepsilon_{1}p_{1}\Gamma K_{2}K_{3}\gamma \lambda_{P}^{*}}{K_{1}{K_{2}}^{2}{K_{3}}^{2}\left(\lambda_{P}^{*}+\mu \right)-K_{1}K_{2}K_{3}\eta \gamma \lambda_{P}^{*}}, \end{array}$$

where *K*_1_ = *ε*_1_ + *μ*, *K*_2_ = *γ* + *μ* + *μ*_2_, and *K*_3_ = *μ* + *η*.

We substitute *P*_*I*_^∗^ stated in equation (21) in equation (20), we computed as *N*_2_^∗^*λ*_*P*_^∗^ = *β*_2_*P*_*I*_^∗^ and gives us the result

(22) $$\begin{array}{c} \left(1-p_{1}\right)\Gamma K_{1}{K_{2}}^{2}{K_{3}}^{2}+\varepsilon_{1}p_{1}\Gamma {K_{2}}^{2}{K_{3}}^{2}+p_{1}\Gamma {K_{2}}^{2}{K_{3}}^{2}\lambda_{P}^{*}+p_{1}\Gamma {K_{2}}^{2}{K_{3}}^{2}\mu +\left(1-p_{1}\right)\Gamma K_{1}K_{2}{K_{3}}^{2}\lambda_{P}^{*}-p_{1}\Gamma K_{2}K_{3}\eta \gamma \lambda_{P}^{*}+\varepsilon_{1}p_{1}\Gamma K_{2}{K_{3}}^{2}\lambda_{P}^{*}+\left(1-p_{1}\right)\Gamma K_{1}K_{2}K_{3}\gamma \lambda_{P}^{*}+\varepsilon_{1}p_{1}\Gamma K_{2}K_{3}\gamma \lambda_{P}^{*}-\beta_{2}\left(1-p_{1}\right)\Gamma K_{1}K_{2}{K_{3}}^{2}-\beta_{2}\varepsilon_{1}p_{1}\Gamma K_{2}{K_{3}}^{2}=0. \end{array}$$

Rearranging (22), we have derived the nonzero linear equation.

(23) $$\begin{array}{c} B_{1}\lambda_{P}^{*}+B_{0}=0. \end{array}$$

where

(24) $$\begin{array}{c} B_{1}=p_{1}\Gamma K_{2}K_{3}\left(K_{2}K_{3}-\eta \gamma \right)+\left(1-p_{1}\right)\Gamma K_{1}K_{2}K_{3}\left(K_{3}+\gamma \right)+\varepsilon_{1}p_{1}\Gamma K_{2}K_{3}\left(K_{3}+\gamma \right)>0, \end{array}$$

(25) $$\begin{array}{c} B_{0}=\Gamma {K_{2}K_{3}}^{2}\left(\left(1-p_{1}\right)K_{1}K_{2}+\varepsilon_{1}p_{1}K_{2}\right)\left[1-R_{P}^{0}\right]<0 \end{array}$$

if and only if *ℛ*_*P*_^0^ > 1 since each parameter has a positive value. Computing the expression in equation (23), we have obtained the result given by

(26) $$\begin{array}{c} \lambda_{P}^{*}=-\frac{B_{0}}{B_{1}}=\frac{\Gamma {K_{2}K_{3}}^{2}\left(p_{2}K_{1}K_{2}+\varepsilon_{1}p_{1}K_{2}\right)\left[R_{P}^{0}-1\right]}{p_{1}\Gamma K_{2}K_{3}\left(K_{2}K_{3}-\eta \gamma \right)+p_{2}\Gamma K_{1}K_{2}K_{3}\left(K_{3}+\gamma \right)+\varepsilon_{1}p_{1}\Gamma K_{2}K_{3}\left(K_{3}+\gamma \right)}>0 \end{array}$$

if and only if *ℛ*_*P*_^0^ > 1 since each of the parameters is positive. Thus, the pneumonia submodel given in equation (19) has a unique positive endemic equilibrium point only whenever *ℛ*_*P*_^0^ > 1.

**Lemma 4 .**

The pneumonia submodel given in equation (19) has a unique positive endemic equilibrium if and only if *ℛ*_*P*_^0^ > 1.

#### 3.3.3. Global Asymptotic Stability of Disease-Free Equilibrium Point

**Lemma 5 .**

The pneumonia submodel (19) disease-free equilibrium point given by the expression

*E*_*P*_^0^ = ( (1 − *p*_1_)Γ(*ε*_1_ + *μ*) + *ε*_1_*p*_1_Γ/*μ*(*ε*_1_ + *μ*), (*p*_1_Γ/*ε*_1_ + *μ*), 0, 0) is globally asymptotically stable if and only if *ℛ*_*P*_^0^ < 1 and the two sufficient conditions given in Lemma 4 holds.

**Proof**

Using the criteria stated by Lemma 4 above on the pneumonia submodel (19) and setting *X* ∈ ℝ^2^ be the components of noninfected individuals and *V* ∈ ℝ^2^ be the components of infected individuals including recovery class. Then, we have determined the following matrices:

(27) $$\begin{array}{c} \frac{dX}{dt}=I\left(X,V\right)=\left[\begin{array}{c} \left(1-p_{1}\right)\Gamma +\varepsilon_{1}P_{P}+\eta {\text{P}}_{T}-\left(\lambda_{P}+\mu \right)S\\ p_{1}\Gamma -\left(\varepsilon_{1}+\mu \right)P_{P} \end{array}\right],\\ \frac{dV}{dt}=J\left(X,V\right)=\left[\begin{array}{c} \lambda_{P}S-\left(\gamma +\mu +\mu_{2}\right)P_{I}\\ \gamma P_{I}-\left(\mu +\eta \right)P_{T} \end{array}\right],\\ I\left(X,0\right)=\left[\begin{array}{c} \left(1-p_{1}\right)\Gamma +\varepsilon_{1}P_{P}-\mu S\\ p_{1}\Gamma -\left(\varepsilon_{1}+\mu \right)P_{P} \end{array}\right],\\ B=D_{V}J\left(X^{0},0\right)=\left[\begin{array}{cc} \frac{\beta_{2}S^{0}}{S^{0}+P_{P}^{0}}-\left(\gamma +\mu +\mu_{2}\right) & 0\\ \gamma & -\left(\mu +\eta \right) \end{array}\right]. \end{array}$$

After we perform some calculations, we have determined that

(28) $$\begin{array}{c} \overset{ˇ}{J}\left(X,V\right)=\left[\begin{array}{c} {\overset{ˇ}{J}}_{1}\left(X,V\right)\\ {\overset{ˇ}{J}}_{2}\left(X,V\right) \end{array}\right]=\begin{array}{c} \left[\begin{array}{c} \frac{\beta_{2}S^{0}P_{I}}{S^{0}+P_{P}^{0}}-\frac{\beta_{2}P_{I}S}{N_{2}}\\ 0 \end{array}\right] \end{array}=\left[\begin{array}{c} \beta_{2}P_{I}\left(\frac{S^{0}}{S^{0}+P_{P}^{0}}-\frac{S}{N_{2}}\right)\\ 0 \end{array}\right]. \end{array}$$

Since ≤*S*^0^, *P*_*P*_ < *P*_*P*_^0^, one can show that *S* − *S*^0^ ≤ 1, *P*_*P*_ − *P*_*P*_^0^ ≤ 1, and$\;{\overset{ˇ}{J}}_{1}\left(X,V\right)\geq 0$; thus, the disease-free equilibrium point *E*_*P*_^0^ = ( (1 − *p*_1_)Γ(*ε*_1_ + *μ*) + *ε*_1_*p*_1_Γ/*μ*(*ε*_1_ + *μ*), (*p*_1_Γ/*ε*_1_ + *μ*), 0, 0) of the pneumonia monoinfection model (19) is globally asymptotically stable if *ℛ*_*P*_^0^ < 1. Epidemiologically, it means whenever *ℛ*_*P*_^0^ < 1, the pneumonia-only disease dies out while the total population increases.

### 3.4. Qualitative Analysis of Pneumonia and HIV/AIDS Coinfection Model

In Sections 3.2 and 3.3, we analyzed the HIV/AIDS and pneumonia single infection models, respectively, and based on the results on these submodels now considered and analyzed the full HIV/AIDS and pneumonia coinfection model in the bounded region *Ω* illustrated in equation (7).

#### 3.4.1. Stability of Disease-Free Equilibrium Point

The full coinfection model (4) disease-free equilibrium point is computed by setting each of the equations in the model equal to zero in the absence of infections and treatment such that *H*_*I*_ = *H*_*A*_ = *P*_*I*_ = C = *P*_*T*_ = *T*_*HA*_ = 0. Thus, after some calculations, we have determined the HIV/AIDS and pneumonia coinfection disease-free equilibrium point given by

(29) $$\begin{array}{c} \;E_{\text{HP}}^{0}=\left({\text{S}}^{0},P_{P}^{0},H_{P}^{0},H_{I}^{0},H_{A}^{0},P_{I}^{0},\;{\text{C}}^{0},{\text{P}}_{\text{T}}^{0},T_{\text{HA}}^{0}\right)=\frac{\left(1-p_{1}-p_{2}\right)\Gamma \left(\varepsilon_{1}+\mu \right)\left(\varepsilon_{2}+\mu \right)+\varepsilon_{1}p_{1}\Gamma +\varepsilon_{2}p_{2}\Gamma \left(\varepsilon_{1}+\mu \right)}{\mu \left(\varepsilon_{1}+\mu \right)\left(\varepsilon_{2}+\mu \right)},\frac{\varepsilon_{1}\Gamma }{\varepsilon_{1}+\mu },\frac{p_{2}\Gamma }{\varepsilon_{2}+\mu },\left.0,0,0,0,0,0\right). \end{array}$$

Similarly, using the same criteria stated in [22], the coinfection model (4) basic reproduction number denoted by *ℛ*_HP_^0^is to be determined as

(30) $$\begin{array}{c} \text{F}{\text{V}}^{-1}=\left[\begin{array}{cccccc} \frac{\beta_{1}\left(\mu \left(1-p_{2}\right)+\alpha_{2}\right)}{\left(\mu +\xi_{1}+\tau \right)\left(\mu +\varepsilon_{2}\right)}+\frac{\beta_{1}\mu \alpha \left(1-p_{2}\right)+\left.\beta_{1}\varepsilon_{2}\alpha \right)}{\left(\mu +\mu_{1}+\xi_{2}\right)\left(\mu +\varepsilon_{2}\right)} & 0 & 0 & 0 & 0 & 0\\ 0 & \frac{\beta_{2}\left(\mu \left(1-p_{1}\right)+\varepsilon_{1}\right)}{\left(\mu +\mu_{2}+\gamma \right)\left(\mu +\varepsilon_{1}\right)} & 0 & 0 & 0 & 0\\ 0 & 0 & 0 & 0 & 0 & 0\\ 0 & 0 & 0 & 0 & 0 & 0\\ 0 & 0 & 0 & 0 & 0 & 0\\ 0 & 0 & 0 & 0 & 0 & 0 \end{array}\right]. \end{array}$$

The coinfection model (4) basic reproduction number is the dominant eigenvalue in magnitude of the next generation matrix *F*.*V*^−1^  given by

(31) $$\begin{array}{c} R_{\text{HP}}^{0}=\max\left\{R_{H}^{0},R_{P}^{0}\right\}=\max\left\{\frac{\beta_{1}\left(\mu \left(1-p_{2}\right)+\alpha_{2}\right)}{\left(\mu +\xi_{1}+\tau \right)\left(\mu +\varepsilon_{2}\right)}+\frac{\beta_{1}\mu \alpha \left(1-p_{2}\right)+\left.\beta_{1}\varepsilon_{2}\alpha \right)}{\left(\mu +\mu_{1}+\xi_{2}\right)\left(\mu +\varepsilon_{2}\right)},\frac{\beta_{2}\left(\mu \left(1-p_{1}\right)+\varepsilon_{1}\right)}{\left(\mu +\mu_{2}+\gamma \right)\left(\mu +\varepsilon_{1}\right)}\right\}, \end{array}$$

where *ℛ*_*H*_^0^ represent the HIV/AIDS-only basic reproduction number, *ℛ*_*P*_^0^ represents the pneumonia only basic reproduction number, and *ℛ*_*HP*_^0^ represents the coinfection basic reproduction numbers, respectively.

In the similar manner of the single infections, the basic reproduction number of HIV/AIDS and pneumonia coinfection is defined as the estimated number of secondary infectious produced by one coinfected individual during its entire period of infectiousness in the whole susceptible population, and the disease-free equilibrium point given by

(32) $$\begin{array}{c} \;E_{\text{HP}}^{0}=\left({\text{S}}^{0},P_{P}^{0},H_{P}^{0},H_{I}^{0},H_{A}^{0},P_{I}^{0},{\text{C}}^{0},{\text{P}}_{\text{T}}^{0},T_{HA}^{0}\right)=\frac{\left(1-p_{1}-p_{2}\right)\Gamma \left(\varepsilon_{1}+\mu \right)\left(\varepsilon_{2}+\mu \right)+\varepsilon_{1}p_{1}\Gamma +\varepsilon_{2}p_{2}\Gamma \left(\varepsilon_{1}+\mu \right)}{\mu \left(\varepsilon_{1}+\mu \right)\left(\varepsilon_{2}+\mu \right)},\frac{\varepsilon_{1}\Gamma }{\varepsilon_{1}+\mu },\frac{p_{2}\Gamma }{\varepsilon_{2}+\mu },\left.0,0,0,0,0,0\right) \end{array}$$

is locally asymptotically stable if and only if *ℛ*_HP_^0^ < 1 and unstable if *ℛ*_HP_^0^ > 1.

#### 3.4.2. Endemic Equilibrium of the Model (4)

The full coinfection model (4) endemic equilibrium points are determined by setting each differential equation equal to zero, and we obtained the result given by

(33) $$\begin{array}{c} S^{*}=\frac{\left(1-p_{1}-p_{2}\right)\Gamma +\varepsilon_{1}P_{P}^{*}+\varepsilon_{2}H_{P}^{*}+\eta P_{T}^{*}}{\left(\lambda_{H}^{*}+\lambda_{P}^{*}+\mu \right)},\\ P_{P}^{*}=\frac{p_{1}\Gamma }{\left(\delta \lambda_{H}^{*}+\varepsilon_{1}+\mu \right)},\\ \;H_{P}^{*}=\frac{p_{2}\Gamma }{\left(\delta \varepsilon_{2}+\mu +\sigma \lambda_{P}^{*}\right)},\\ \;H_{I}^{*}=\frac{\lambda_{H}^{*}S^{*}+\delta \lambda_{H}^{*}P_{P}^{*}}{\left(\mu +\xi_{1}+\tau +\phi_{1}\lambda_{P}^{*}\right)},\\ H_{A}^{*}=\frac{\tau H_{I}^{*}}{\left(\mu +\mu_{1}+\xi_{2}+\phi_{2}\lambda_{P}^{*}\right)},\\ \;P_{I}^{*}=\frac{\lambda_{P}^{*}S^{*}+\delta \lambda_{P}^{*}H_{P}^{*}}{\left(\gamma +\mu +\mu_{2}+\varphi \lambda_{H}^{*}\right)},\\ \;C^{*}=\frac{\varphi \lambda_{H}^{*}P_{I}^{*}+\phi_{1}\lambda_{P}^{*}H_{I}^{*}+\phi_{2}\lambda_{P}^{*}H_{A}^{*}+\rho \lambda_{P}^{*}T_{HA}^{*}}{\left(\mu +\mu_{3}+\theta \right)},\\ \;P_{T}^{*}=\frac{\gamma P_{I}^{*}}{\left(\mu +\eta \right)},\\ T_{HA}^{*}=\frac{\xi_{1}\;H_{I}^{*}+\xi_{2}\;H_{A}^{*}+\theta C^{*}}{\left(\rho \lambda_{P}^{*}+\mu \right)}. \end{array}$$

The coinfection model (4) we proposed is highly nonlinear, and hence, the explicit computation of the endemic equilibrium point(s) in terms of the illustrated model parameters is difficult analytically; however, based on the previous analyses of the HIV/AIDS and pneumonia submodels, the endemic equilibrium point(s) represented by *E*_HP_^∗^ = (*S*^∗^, *P*_*P*_^∗^, *H*_*P*_^∗^, *H*_*I*_^∗^, *H*_*A*_^∗^, *P*_*I*_^∗^, *C*^∗^, *P*_*T*_^∗^, *T*_HA_^∗^) exists whenever  *ℛ*_*H*_^0^ > 1 and *ℛ*_*P*_^0^ > 1, i.e., *ℛ*_*HP*_^0^ > 1. The stability is shown in the numerical simulation part.

#### 3.4.3. Possibility of Existence of Backward Bifurcation for the Coinfection Dynamical System (4)

Let *S* = *v*_1_, *P*_*P*_ = *v*_2_, *H*_*P*_ = *v*_3_, *H*_*I*_ = *v*_4_, *H*_*A*_ = *v*_5_, *P*_*I*_ = *v*_6_, *C* = *v*_7_, *P*_*T*_ = *v*_8_, and *T*_*HA*_ = *v*_9_, and the total human population is given by *N* = *v*_1_ + *v*_2_ + *v*_3_ + *v*_4_ + *v*_5_, +*v*_6_ + *v*_7_, +*v*_8_ + *v*_9_.

Moreover, by the vector representation *V* = (*v*_1_, *v*_2_, *v*_3_, *v*_4_, *v*_5_, *v*_6_, *v*_7_, *v*_8_, *v*_9_)^*T*^, the dynamical system (4) will be rewritten as (*dV*/*dt*) = *H*(*V*) with *H* = (*h*_1_, *h*_2_, *h*_3_, *h*_4_, *h*_5_, *h*_6_, *h*_7_, *h*_8_, *h*_9_)^*T*^ and

(34) $$\begin{array}{c} \frac{{dv}_{1}}{dt}=h_{1}=\left(1-p_{1}-p_{2}\right)\Gamma +\varepsilon_{1}v_{2}+\varepsilon_{2}v_{3}+\eta v_{8}-\left(\lambda_{H}+\lambda_{P}+\mu \right)v_{1},\\ \frac{{dv}_{2}}{dt}=h_{2}=p_{1}\Gamma -\left(\delta \lambda_{H}+\varepsilon_{1}+\mu \right)v_{2},\\ \frac{{dv}_{3}}{dt}=h_{3}=p_{2}\Gamma -\left(\varepsilon_{2}+\mu +\sigma \lambda_{P}\right)v_{3},\\ \frac{{dv}_{4}}{dt}=h_{4}=\lambda_{H}v_{1}+\delta \lambda_{H}v_{2}-\left(\mu +\tau +\xi_{1}+\phi_{1}\lambda_{P}\right)v_{4},\\ \frac{{dv}_{5}}{dt}=h_{5}=\tau v_{4}-\left(\mu +\mu_{1}+\xi_{2}+\phi_{2}\lambda_{P}\right)v_{5},\\ \frac{{dv}_{6}}{dt}=h_{6}=\lambda_{P}v_{1}+\sigma \lambda_{P}v_{3}-\left(\gamma +\mu +\mu_{2}+\varphi \lambda_{H}\right)v_{6},\\ \frac{{dv}_{7}}{dt}=h_{7}=\varphi \lambda_{H}v_{6}+\phi_{1}\lambda_{P}v_{4}+\phi_{2}\lambda_{P}v_{5}+\rho \lambda_{P}v_{9}-\left(\mu +\mu_{3}+\theta \right)v_{7},\\ \frac{{dv}_{8}}{dt}=h_{8}=\gamma v_{6}-\left(\mu +\eta \right)v_{8},\\ \frac{{dv}_{9}}{dt}=h_{9}=\xi_{1}v_{4}+\xi_{2}v_{5}+\theta v_{7}-\rho \lambda_{P}v_{9}-\mu v_{9}, \end{array}$$

where *λ*_*H*_ = (*β*_1_/*N*)[*v*_4_ + *αv*_5_ + *ϑv*_7_] for 1 ≤ *ρ*_1_ < ∞ and *λ*_*P*_ = (*β*_2_/*N*)[*v*_6_ + *ωv*_7_] for 1 ≤ *ω* < ∞.

Then, the Jacobian matrix of the new dynamical system given in (22) at *E*_HP_^0^, represented by *J*(*E*_HP_^0^) and determined by

(35) $$\begin{array}{c} J\left(E_{\text{HP}}^{0}\right)=\left(\begin{array}{ccccccccc} -\mu & \;\varepsilon_{1} & \;\varepsilon_{2} & \;E_{1} & \;E_{2} & E_{3} & E_{4} & \eta & 0\\ 0 & -\left(\varepsilon_{1}+\mu \right) & 0 & \;E_{5} & \;E_{6} & 0 & E_{7} & 0 & 0\\ 0 & 0 & -\left(\varepsilon_{2}+\mu \right) & 0 & 0 & E_{8} & E_{9} & 0 & 0\\ 0 & 0 & 0 & E_{10} & E_{11} & 0 & E_{12} & 0 & 0\\ 0 & 0 & 0 & \tau & -\left(\mu +\mu_{1}+\xi_{2}\right) & 0 & 0 & 0 & 0\\ 0 & 0 & 0 & 0 & 0 & E_{13} & E_{14} & 0 & \;0\\ 0 & 0 & 0 & 0 & 0 & 0 & -\left(\mu +\mu_{3}+\theta \right) & 0 & 0\\ 0 & 0 & 0 & 0 & 0 & \gamma & 0 & -\left(\mu +\eta \right) & 0\\ 0 & 0 & 0 & \xi_{1} & \xi_{2} & 0 & \theta & 0 & -\mu \end{array}\right), \end{array}$$

where *E*_1_ = −(*β*_1_/*N*^0^)*v*_1_^0^, *E*_2_ = −(*β*_1_*α*/*N*^0^)*v*_1_^0^, *E*_3_ = −(*β*_2_/*N*^0^)*v*_1_^0^, *E*_4_ = −(*β*_1_/*N*^0^)*ϑv*_1_^0^ − (*β*_2_/*N*^0^)*ωv*_1_^0^, *E*_5_ = −(*β*_1_/*N*^0^)*v*_2_^0^, *E*_6_ = (*β*_1_/*N*^0^)*δαv*_2_^0^, *E*_7_ = −(*β*_1_/*N*^0^)*ρ*_1_*v*_2_^0^, *E*_8_ = −(*β*_2_/*N*^0^)*v*_3_^0^, *E*_9_ = −(*β*_2_/*N*^0^)*ωv*_3_^0^,  *E*_10_ = (*β*_1_/*N*^0^)*v*_1_^0^ + (*β*_1_/*N*^0^)*δv*_2_^0^ − (*μ* + *τ* + *ξ*_1_),  *E*_11_ = (*β*_1_/*N*^0^)*αv*_1_^0^ + (*β*_1_/*N*^0^)*δv*_2_^0^, *E*_12_ = (*β*_1_/*N*^0^)*ϑv*_1_^0^ + (*β*_1_/*N*^0^)*δϑv*_2_^0^, *E*_13_ = (*β*_2_/*N*^0^)*v*_1_^0^ + (*β*_2_/*N*^0^)*v*_3_^0^ − (*γ* + *μ* + *μ*_2_), *E*_14_ = (*β*_2_/*N*^0^)*ωv*_1_^0^ + (*β*_2_/*N*^0^)*ωv*_3_^0^.

Let us assume *ℛ*_*P*_^0^ > *ℛ*_*H*_^0^ without loss of the generality, and *ℛ*_HP_^0^ = 1, i.e., *ℛ*_*P*_^0^ = 1. Moreover, let *β*_2_ = *β*^∗^ be a bifurcation parameter. Solving for *β*_2_ using *ℛ*_*P*_^0^ = 1 as *ℛ*_*P*_^0^ = (*β*_2_(*μ*(1 − *p*_1_) + *ε*_1_)/(*μ* + *μ*_2_ + *γ*)(*μ* + *ε*_1_)) = 1, we determined as *β*^∗^ = *β*_2_ = ((*μ* + *μ*_2_ + *γ*)(*μ* + *ε*_1_)/(*μ*(1 − *p*_1_) + *ε*_1_)).

Then, we compute the eigenvalues of the Jacobian matrix *J*(*E*_HP_^0^) at *E*_HP_^0^, for *β*_2_ = *β*^∗^, and we determined the eigenvalues given by *λ*_1_ = −*μ*<0 or *λ*_2_ = −(*ε*_1_ + *μ*) < 0 or *λ*_3_ = −(*ε*_2_ + *μ*) < 0 or *λ*_4_ = *E*_10_ = (*β*_1_/*N*^0^)*v*_1_^0^ + (*β*_1_/*N*^0^)*δv*_2_^0^ − (*μ* + *τ* + *ξ*_1_) = (*μ* + *τ* + *ξ*_1_)[*ℛ*_*H*_^0^ − 1] < 0 if *ℛ*_*H*_^0^ < 1 or *λ*_5_ = 0 or *λ*_6_ = *E*_13_ = (*β*_2_/*N*^0^)*v*_1_^0^ + (*β*_2_/*N*^0^)*v*_3_^0^ − (*γ* + *μ* + *μ*_2_) = (*γ* + *μ* + *μ*_2_)[*ℛ*_*P*_^0^ − 1] < 0 if *ℛ*_*P*_^0^ < 1 or *λ*_7_ = −(*μ* + *d*_3_ + *θ*) < 0 or *λ*_8_ = −(*μ* + *η*) < 0 or *λ*_9_ = −*μ* < 0. From the computations, we observed that all the eigenvalues are negative if *ℛ*_HP_^0^ < 1. We apply the centre manifold theory stated in [31], to illustrate that the dynamical system (4) undergoes the phenomenon of forward bifurcation at *ℛ*_*P*_^0^ = 1. For the eigenvectors of the Jacobian *J*_*β*^∗^_, for the case *ℛ*_*P*_^0^ = 1, the right eigenvectors at *β*_2_ = *β*^∗^ corresponding to the zero eigenvalue given by *y* = (*y*_1_, *y*_2_, *y*_3_, *y*_4_, *y*_5_, *y*_6_, *y*_7_, *y*_8_, *y*_9_)^*T*^ are

(36) $$\begin{array}{c} y_{1}=\frac{\varepsilon_{2}E_{7}\left(\mu +\eta \right)y_{5}+\left(\varepsilon_{2}+\mu \right)\left(\mu +\eta \right)E_{2}y_{5}+\left(\varepsilon_{2}+\mu \right)\eta \gamma y_{5}}{\mu \left(\varepsilon_{2}+\mu \right)\left(\mu +\eta \right)},y_{2}=0,y_{3}=\frac{E_{7}}{\varepsilon_{2}+\mu }y_{5},y_{4}=0,y_{5}=y_{5}>0,y_{6}=0,y_{7}=\frac{\kappa }{\mu +\eta }u_{5},y_{8}=0,y_{8}=0. \end{array}$$

Left eigenvectors corresponding to the zero eigenvalue at *β*_2_ = *β*_2_^∗^ that holds *y*.*z* = 1, given by

(37) $$\begin{array}{c} z=\left(z_{1},z_{2},z_{3},z_{4},z_{5},z_{6},z_{7},z_{8},z_{9}\right)\text{ and }z_{1}=z_{2}=z_{3}=z_{4}=z_{6}=z_{7}=z_{8}=z_{9}=0\text{ and }z_{5}=z_{5}>0. \end{array}$$

After many steps of calculations and simplification, we determined the bifurcation coefficients given by *a* and *b* as

(38) $$\begin{array}{c} a=2z_{5}y_{1}y_{5}\frac{\partial^{2}h_{5}\left(0,0\right)}{\partial v_{1}\partial v_{5}}+2z_{5}y_{3}y_{5}\frac{\partial^{2}h_{5}\left(0,0\right)}{\partial v_{2}\partial v_{5}}=2\beta_{2}^{*}z_{5}y_{5}\left[y_{1}+y_{3}\right],\\ =2\beta_{2}^{*}z_{5}y_{5}^{2}\left[\frac{-\varepsilon_{2}\beta_{2}v_{3}^{0}\left(\mu +\eta \right)-\left(\varepsilon_{2}+\mu \right)\left(\mu +\eta \right)\beta_{2}v_{1}^{0}-\left(\varepsilon_{2}+\mu \right)\eta \gamma -\mu \left(\mu +\eta \right)\beta_{2}v_{3}^{0}}{\mu \left(\varepsilon_{2}+\mu \right)\left(\mu +\eta \right)}\right]. \end{array}$$

Thus,

(39) $$\begin{array}{c} a=-2\beta_{2}^{*}z_{5}y_{5}^{2}\left[\frac{\varepsilon_{2}\beta_{2}y_{3}^{0}\left(\mu +\eta \right)+\left(\varepsilon_{2}+\mu \right)\left(\mu +\eta \right)\beta_{2}v_{1}^{0}+\left(\varepsilon_{2}+\mu \right)\eta \gamma +\mu \left(\mu +\eta \right)\beta_{2}v_{3}^{0}}{\mu \left(\alpha_{2}+\mu \right)\left(\mu +\eta \right)}\right]<0,\\ b=z_{5}y_{5}\frac{\partial^{2}h_{5}\left(0,0\right)}{\partial {\text{v}}_{5}\partial \beta_{2}}=z_{5}y_{5}\left(v_{3}^{0}+v_{1}^{0}\right)>0. \end{array}$$

Therefore, using the criteria stated in [31], the HIV/AIDS and pneumonia coinfection dynamical system (4) do not exhibit the phenomenon of backward bifurcation whenever *ℛ*_HP_^0^ = *ℛ*_*P*_^0^ = 1. Thus, there is no positive endemic equilibrium point rather there is only the coinfection model disease-free equilibrium point in the region at which *ℛ*_HP_^0^ < 1.

## 4. Sensitivity and Numerical Analysis

In this section, we need to verify the qualitative analysis results performed in Section 3, and we have performed several sensitivity and numerical analyses. In this study, to obtain more relevant model parameters illustrated in Table 3, we have observed and reviewed different research studies based on the mathematical modelling on infectious diseases, and for some other parameters, we assumed realistic values for the purpose of sensitivity and numerical analyses and illustrations.

### 4.1. The Coinfection Model Sensitivity Analysis

**Definition 1 .**

Let *z* be variable; then, the normalized forward sensitivity index of *z* which depends differentially on a parameter *ϑ* is defined as SEI *D*(*ϑ*) = (*∂z*/*∂ϑ*)∗(*ϑ*/*z*) [27].

The sensitivity indices we have calculated in this subsection allow to investigate the relative significance of various parameters in the proposed HIV/AIDS and pneumonia coinfection spreading dynamics. The parameter which has larger magnitude than that of all other parameters is the most sensitive parameter. Now, we can compute the sensitivity indices in terms of the model basic reproduction numbers *ℛ*_*H*_^0^  and *ℛ*_*P*_^0^  since *ℛ*_HP_^0^ = max{*ℛ*_*H*_^0^, *ℛ*_*P*_^0^}.

Applying the baseline parameter values given in Table 3, we have derived Tables 4 and 5 to show the sensitivity indices of the model parameters.

In this study, with the baseline parameter values given in Table 3, we have computed *ℛ*_*H*_^0^ = 1.91 which implies that HIV/AIDS spreads in the community, and we also have determined the indices in Table 4. Sensitivity analysis results show that the HIV/AIDS spreading rate *β*_1_ has the highest impact on the HIV/AID only infection basic reproduction number (*ℛ*_*H*_^0^).

Similarly, using baseline parameter values given in Table 3, we have computed *ℛ*_*P*_^0^ = 3.86 which implies that pneumonia is spreading throughout the community, and we also have computed the sensitivity indices as shown in Table 5. Sensitivity analysis results show that the foremost sensitive positive parameter is the pneumonia spreading rate *β*_2_. Using Tables 4 and 5, biologically, we can conclude that the most sensitive parameters are the HIV/AIDS and pneumonia spreading rates.

**Figure 2:**
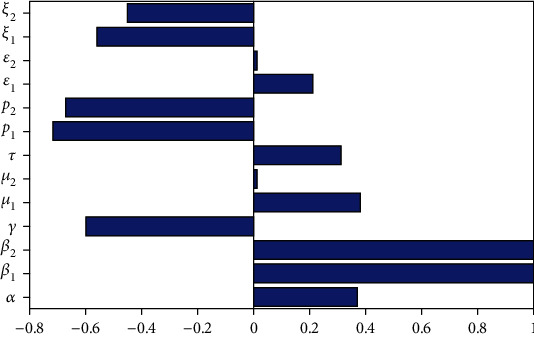
Simulation of sensitivity indices of parameters with respect to *ℛ*_HP_^0^.

In this subsection, we performed numerical simulation illustrated in Figure 2 to investigate the HIV/AIDS and pneumonia coinfection model parameters sensitivity indices with respect to the coinfection reproduction number, and from the result, we observed that both the HIV and pneumonia spreading rates *β*_1_ and *β*_2_, respectively, are epidemiologically the most sensitive parameters having a direct proportionality with the HIV/AIDS and pneumonia reproduction numbers, respectively. Furthermore, the HIV infection protection portion (*p*_2_) and pneumonia infection protection portion (*p*_2_) and treatment rates are more sensitive parameters having an indirect proportionality with the associated reproduction number.

### 4.2. The Coinfection Model Numerical Simulations

In this part, we carried out simulations for the HIV/AIDS and pneumonia codynamics by using the parameter baseline values given in Table 3 mainly to verify the qualitative analysis performed throughout Section 3. To investigate the numerical results of the constructed coinfection model (4), the initial data should have nonnegative values because the number of people in each class cannot be negative. In this subsection, the numerical simulations were conducted with MATLAB by applying the Runge-Kutta ODE45 method. Throughout this subsection, we examine the behavior of the coinfection model solutions and their convergence to the corresponding equilibrium points, investigate the impact of the model parameters on the diseases spreading in the community, and more specifically examine the effect of protection and treatment strategies on the diseases spreading dynamics. In order to simulate the HIV/AIDS and pneumonia coinfection model (4), set the nonnegative initial data (S(0), *P*_*P*_(0), *H*_*P*_(0), *H*_*I*_(0), *H*_*A*_(0), *P*_*I*_(0), C(0), P_T_(0), *T*_*HA*_(0)) = (1500, 350, 250, 150, 100, 200,90,85,70).

#### 4.2.1. Simulation to Show Behaviour Solutions Whenever *ℛ*_HP_^0^ < 1

**Figure 3:**
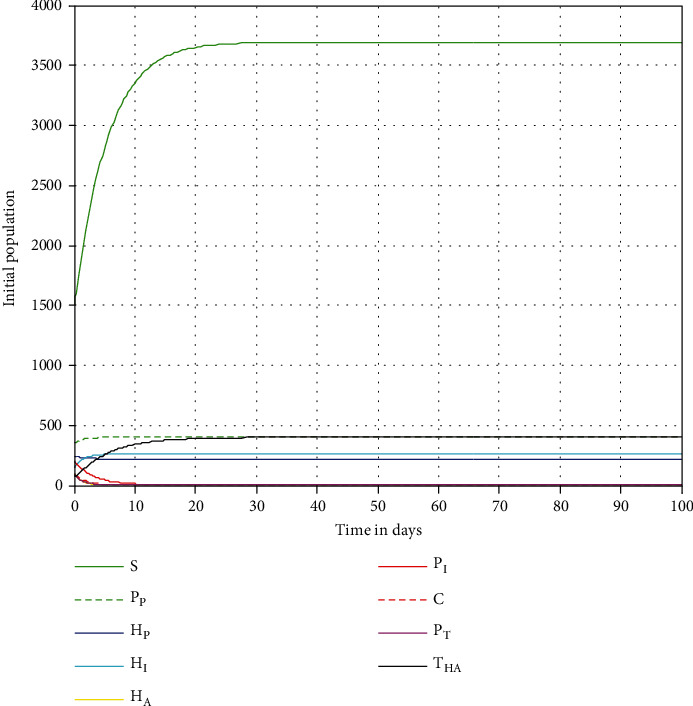
Simulation of the dynamical system (4) solutions at *ℛ*_HP_^0^ = 0.46 < 1.

The numerical trajectories given in Figure 3 show the behavior of the coinfection model solutions over time whenever  *ℛ*_HP_^0^ < 1. From this numerical result, we can justify the qualitative results proved in Section 3.3.1. The HIV/AIDS and pneumonia dynamical system (4) effective reproduction number is calculated as *ℛ*_HP_^0^ = 0.46. We also observed that after 100 days, the coinfection dynamical system solutions converge to the disease-free equilibrium point if  *ℛ*_HP_^0^ = max{*ℛ*_*H*_^0^, *ℛ*_*P*_^0^ } = max{0.46, 0.87 } = 0.87 < 1. Epidemiologically, it means that the coinfection outbreaks in the population will be eliminated in the near future.

#### 4.2.2. Simulation to Show Solution Trajectories Whenever *ℛ*_HP_^0^ > 1

**Figure 4:**
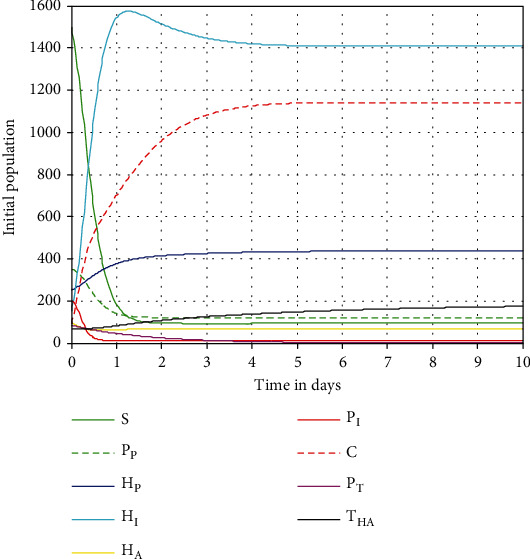
Simulation of the coinfection system (4) at *ℛ*_HP_^0^ = 3.86 > 1.

In this subsection, we have carried out the numerical simulation of the coinfection dynamical to examine the solution trajectory behavior whenever *ℛ*_HP_^0^ = 3.86 > 1.  Figure 4 shows that the simulation trajectories will converge to the model endemic equilibrium point whenever the coinfection model computed effective reproduction number is *ℛ*_HP_^0^ = max{*ℛ*_*H*_^0^, *ℛ*_*P*_^0^} = max{1.91, 3.86 } = 3.86 > 1. It means that the HIV/AIDS and pneumonia coinfection model (4) solutions approach to its endemic equilibrium point if *ℛ*_HP_^0^ = 3.86 > 1.

#### 4.2.3. Simulation to Show the Impact of HIV Spreading Rate on Pneumonia Transmission

**Figure 5:**
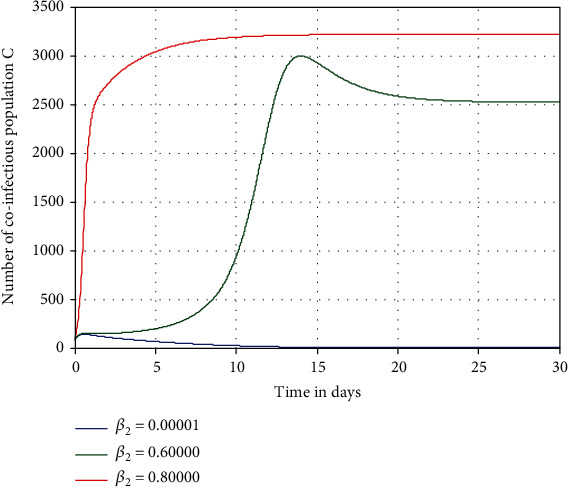
Effect of HIV spreading rate  *β*_1_  on the coinfection *C*.

Numerical simulation illustrated in Figure 5 investigates the impact of HIV spreading rate *β*_1_ on the number of coinfected people denoted by *C*. From the result, we observed that when we increase the value of *β*_1_, then the number of coinfected people in the population increases. Whenever HIV spreading rate *β*_1_ increases   from a value 0.00001 to a value 0.8, then the HIV/AIDS and pneumonia coinfection population denoted by *C* is highly increases, and thus, we recommend for the stakeholders to exert their optimum effort on decrease the HIV spreading rate with applying suitable intervention mechanisms.

#### 4.2.4. Simulation to Investigate the Impact of Pneumonia Spreading on the Coinfection

**Figure 6:**
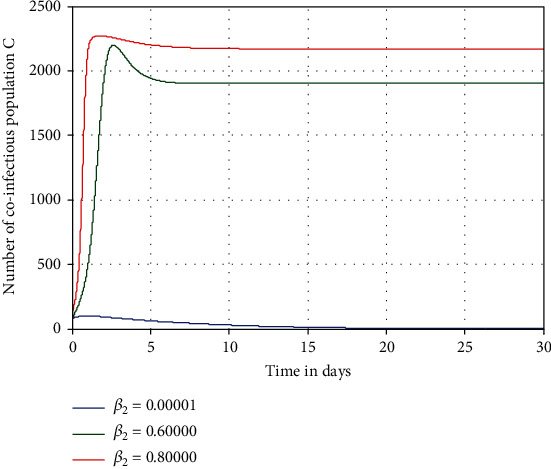
Effect of pneumonia spreading rate *β*_2_ on the coinfection *C*.

Numerical simulation illustrated in Figure 6 investigates the impact of pneumonia spreading rate *β*_2_ on the number of coinfectious people denoted by *C*. From the result, we observed that increasing the value of *β*_2_ leads to an increase of the number of coinfectious people in the population. Consequently, increasing pneumonia transmission rate *β*_2_ from 0.00001 to 0.8 leads to a highly increase of HIV/AIDS and pneumonia coinfection *C*.

#### 4.2.5. Treatment Impact on the Number of HIV-Infected Population

**Figure 7:**
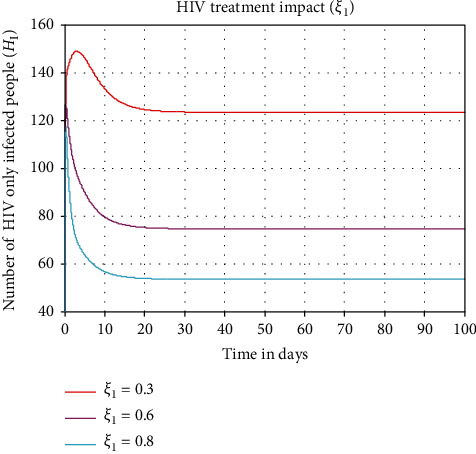
Effect of treatment on HIV-infected population.

In this subsection, we perform numerical simulation illustrated in Figure 7 to investigate the impact of HIV treatment (antiretroviral therapy or ART) rate (*ξ*_1_) on the HIV-infected population denoted by *H*_*I*_. From the numerical simulation result, we observe that whenever we increase the value of HIV treatment (antiretroviral therapy or ART) rate (*ξ*_1_) from 0.3 to 0.8, the number of HIV-infected population is going down throughout the community.

#### 4.2.6. Treatment Impact on the Number of AIDS Patients

**Figure 8:**
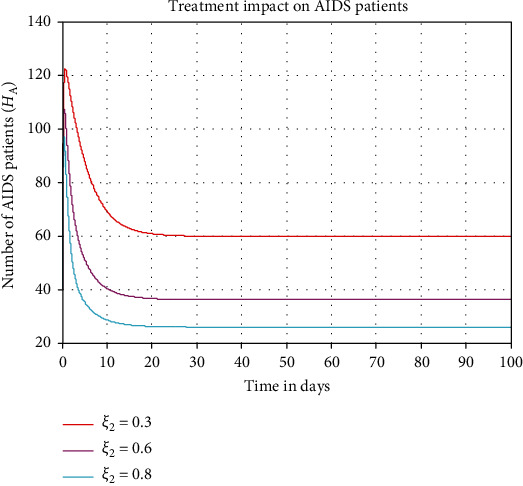
Effect of treatment on AIDS patients.

In this subsection, we perform numerical simulation illustrated in Figure 8 to investigate the impact of HIV treatment (antiretroviral therapy or ART) rate (*ξ*_2_) on the AIDS patient population denoted by *H*_*A*_. From the numerical simulation result, we observe that whenever we increase the value of HIV treatment (antiretroviral therapy or ART) rate (*ξ*_2_) from 0.3 to 0.8, the number of AIDs patient population is going down throughout the community.

#### 4.2.7. Treatment Impact on HIV/AIDS and Pneumonia Coinfection

**Figure 9:**
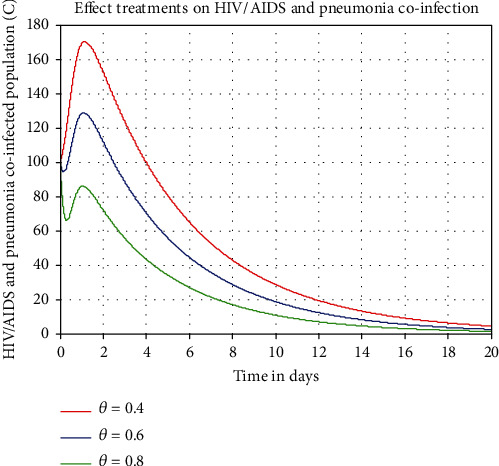
Effect of treatment rate *θ* on the coinfection *C*.

In this part, we simulate the state variable which represents HIV/AIDS and pneumonia coinfection dynamics illustrated in Figure 9. From the result, we observed that whenever the treatment rate *θ* is going up, then the number of HIV/AIDS and pneumonia population decreases in the community. Epidemiologically, it means whenever the stakeholders of human being health increase treatment intervention strategies from the rate 0.4 to the rate 0.8, this implies that the number of HIV/AIDS and pneumonia coinfected individuals is going down.

#### 4.2.8. Impact of the HIV/AIDS Spreading Rate *β*_1_ on *ℛ*_*H*_^0^

**Figure 10:**
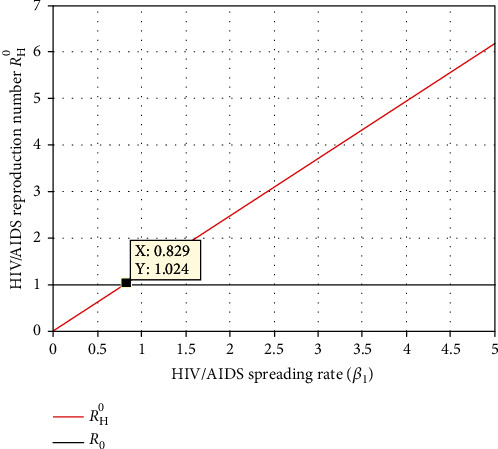
Effect of HIV/AIDS spreading rate *β*_1_ on *ℛ*_*H*_^0^.

Simulation illustrated in Figure 10 investigates the influence of the HIV/AIDS spreading rate *β*_1_ on the effective reproduction number *ℛ*_*H*_^0^. Since increasing the HIV/AIDS spreading rate leads to increase, the HIV/AIDS transmission in the community health stakeholders shall introduce effective intervention strategies to minimize the value of *β*_1_ less than 0.829.

#### 4.2.9. Impact of Portion of Protection against HIV Infection *p*_2_ on *ℛ*_*H*_^0^

**Figure 11:**
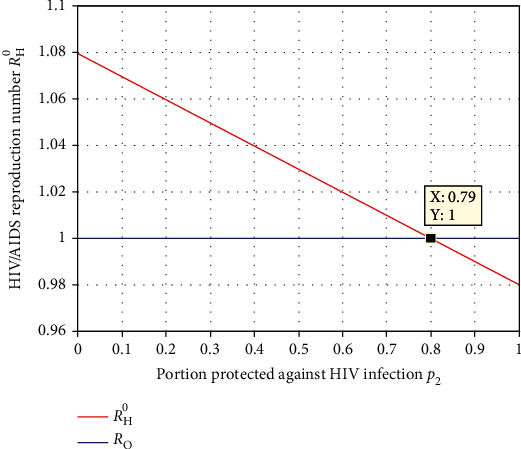
Effect of protection intervention portion  *p*_2_ on *ℛ*_*H*_^0^.

The numerical simulation represented in Figure 11 illustrated that the portion *p*_2_ of the human recruitment rate that entered to the HIV/AIDS protected class using condom intervention strategy has a significant effect on *ℛ*_*H*_^0^. From the result, we observed that increasing the value of *p*_2_ leads to a decrease in the spreading rate of HIV/AIDS in the population. And we recommend for the health stakeholders to introduce the portion of human recruitment portion *p*_2_; more than 0.79 makes the effective reproduction number value *ℛ*_*H*_^0^ below unity.

#### 4.2.10. Impact of HIV Infection Treatment *ξ*_1_ on *ℛ*_*H*_^0^

**Figure 12:**
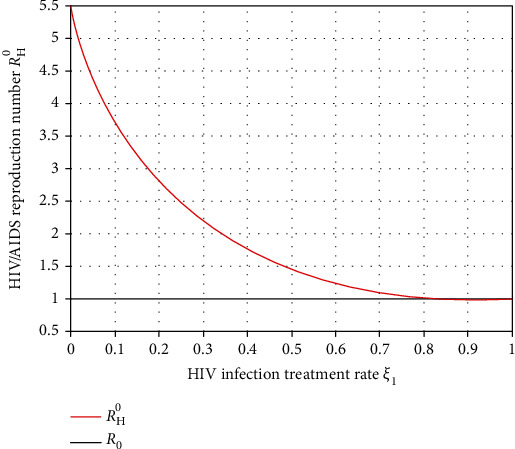
Effect of HIV treatment on *ℛ*_*H*_^0^.

Simulation illustrated in Figure 12 shows that the HIV treatment rate *ξ*_1_ has a significant effect on *ℛ*_*H*_^0^. From the result, we observed that whenever we increase the HIV treatment rate *ξ*_1_, then the HIV spreading rate decreases in the population. We recommend for the health stakeholders to exert their optimum effort to introduce the HIV treatment rate *ξ*_1_ more than the value 0.97 to make *ℛ*_*H*_^0^ less than unity.

#### 4.2.11. Impact of Pneumonia Spreading Rate *β*_2_ on *ℛ*_*P*_^0^

**Figure 13:**
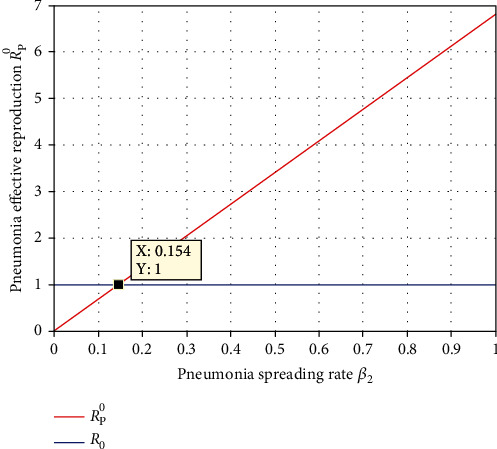
Effect of pneumonia transmission rate *β*_2_ on *ℛ*_*P*_^0^.

Numerical simulation illustrated in Figure 13 examined the effect of pneumonia spreading rate *β*_2_ on the effective reproduction of pneumonia *ℛ*_*P*_^0^. From the figure, we observed that increasing the value of *β*_2_ leads to increase the effective reproduction number of the pneumonia, and whenever *β*_2_ < 0.149 , then *ℛ*_*P*_^0^ < 1. Thus, stakeholders of public health shall exert optimum effort to minimize the spreading rate  *β*_2_ for prevention and controlling of pneumonia spreading throughout the population. Epidemiologically, it means that whenever the pneumonia spreading rate increases, then the pneumonia disease increases in the community, and the disease will be eliminated from the population if  *β*_2_ < 0.154.

#### 4.2.12. Impact of Portion *p*_1_ of Pneumonia Protection on *ℛ*_*P*_^0^

**Figure 14:**
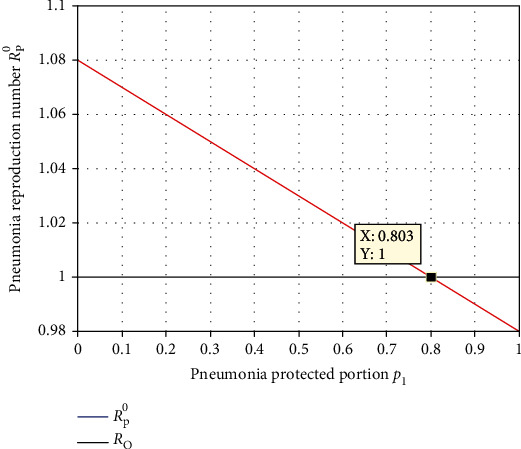
Effect of portion *p*_1_ of pneumonia protection on *ℛ*_*P*_^0^.

Numerical simulation illustrated in Figure 14 shows that the portion *p*_1_ of pneumonia protection of the human recruitment rate has an influential impact on *ℛ*_*P*_^0^. From the result, we observed that increasing the portion of pneumonia protection decreases the pneumonia spreading throughout the population. Thus, for stakeholders, we recommend to introduce the portion *p*_1_ of the human recruitment greater than 0.803 and to make the value of *ℛ*_*P*_^0^ below one.

#### 4.2.13. Impact of Pneumonia Treatment Rate *γ* on *ℛ*_*P*_^0^

**Figure 15:**
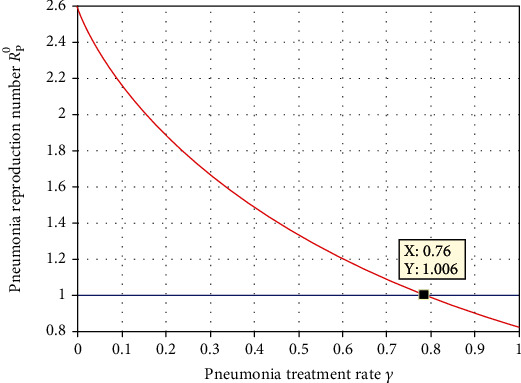
Effect of pneumonia treatment rate *γ* on *ℛ*_*P*_^0^.

Numerical simulation represented in Figure 15 illustrated that the treatment rate *γ* of pneumonia has a crucial indirect role on *ℛ*_*P*_^0^. From the result, we observed that whenever the pneumonia treatment rate increases, then the pneumonia spreading in the population decreases. Thus, we recommend for health stakeholders to exert their optimal effort to maximize treatment rate *γ* more than the value 0.76 and making the value of *ℛ*_*P*_^0^ less than unity.

## 5. Conclusions

This study presented the HIV/AIDS and pneumonia coinfection dynamical system analysis to investigate protection and treatment intervention mechanisms' impacts on the coinfection spreading dynamics. Using parameter values adopted from published literatures, we have determined some basic results from the HIV/AIDS and pneumonia coinfection dynamical system qualitative and numerical analysis stated as follows: the proposed coinfection model has six equilibrium points; the HIV/AIDS submodel disease-free and endemic equilibrium points that are both globally and locally asymptotically stable whenever its effective reproduction number is less than one which indicates that the HIV/AIDS submodel do not exhibits the phenomenon of backward bifurcation; the pneumonia only disease-free and endemic equilibrium points that are both globally and locally asymptotically stable whenever its effective reproduction number is less than one which indicates that the pneumonia submodel do not exhibits the phenomenon of backward bifurcation; and the HIV/AIDS and pneumonia coinfection model disease-free and endemic equilibrium points that are both globally and locally asymptotically stable whenever its effective reproduction number is less than one which indicates that the HIV/AIDS submodel do not exhibits the phenomenon of backward bifurcation. The qualitative and quantitative sensitivity analyses reveal that the disease-spreading rates, protection rates, and treatment rates are the most sensitive parameters at which the stakeholders should give emphasis on these parameters and exert their maximum effort to control the transmission of the diseases in the community by applying suitable intervention measures. The coinfection model numerical simulation performed verified the qualitative results by investigating the impacts of some model parameters on the models associated with effective reproduction, the model state variables, and the behavior of the coinfection model solutions regarding convergence to the model equilibrium points. From the result, we recommend to the health stakeholders to minimize the disease-spreading rates and to maximize the protection and treatment rates for reducing the effective reproduction numbers below one. Finally, since the model formulation in this study is not exhaustive, any potential researcher can modify this study in various ways, such as by incorporating optimal control strategies, stochastic method, fractional order approach, environment effects, age structure, or validating models by collecting real data.

## Figures and Tables

**Table 1:** Parameters used in the model formulation.

Parameter	Interpretation
*μ*	Natural mortality rate
Γ	People recruitment rate
*ε*_1_	Pneumonia protection loss rate
*ε*_2_	HIV protection loss rate
*θ*	HIV/AIDS and pneumonia coinfected people treatment rate
*Φ*_1_, *Φ*_2_	Modification parameters
*φ*	Modification parameter
*μ*_2_	Death rate by pneumonia disease
*μ*_1_	Death rate by AIDS disease
*γ*	Pneumonia treatment rate
*ξ*_1_	HIV-infected treatment rate
*ξ*_2_	AIDS patient treatment rate
*β*_1_	HIV/AIDS spreading rate
*β*_2_	Pneumonia spreading rate
p_1_	Portion of recruitment rate
p_2_	Portion of recruitment rate
*η*	Pneumonia immunity loss rate
*ρ*, *ω*, *φ*, *ϑ*	Modification parameters
*μ*_3_	Death rate by the coinfection

**Table 2:** Variables used in the coinfection model.

Variable	Epidemiological meaning
*S*	Susceptible people
*P*_*P*_	People who are protected against pneumonia
*H*_*P*_	People who are protected against HIV/AIDS
*H*_*I*_	HIV-infected individuals
*H*_*A*_	AIDS patients
*P*_*I*_	Pneumonia-infected people
*C*	People coinfected with HIV/AIDS and pneumonia
*P*_*T*_	People treated from pneumonia
*T*_*HA*_	HIV/AIDS-treated people

**Table 3:** Parameter values used for sensitivity and numerical analyses.

Parameter	Value	Reference
Γ	1000 humans/day	[21]
*μ*	(1/64.5 × 365)/day	[6]
*ε*_1_	0.005/day	[7]
*ε*_2_	0.0004/day	[7]
*μ*_1_	0.00034/day	[7]
*μ*_2_	0. 057/day	[13]
*θ*	0.0021/day	Assumed
*Φ*_1_, *Φ*_2_	1 no unit	Assumed
*δ*, *σ*	1 no unit	Assumed
*ξ*_1_	0.0023/day	[25]
*μ*_3_	0.15/day	Assumed
*φ*	1 no unit	Assumed
*η*	0.1/day	[13]
*β*_1_	0.3425/day	[6]
*β*_2_	0.0115/day	[13]
*p*_1_	0.597/day	[7]
*p*_2_	0.006/day	[7]
*ξ*_2_	0.13/day	[5]
*γ*	0.2/day	[13]
*φ*, *α*, *ω*	1 no unit	Assumed

**Table 4:** Sensitivity indices for *ℛ*_HP_^0^ = *ℛ*_*H*_^0^.

Sensitivity index	Value
SEID(*β*_1_)	+1
SEID(*ε*_2_)	+0.01
SEID(*p*_2_)	-0.67
SEID(*μ*_1_)	+0.38
SEID(*τ*)	+0.31
SEID(*ξ*_1_)	-0.56
SEID(*ξ*_2_)	-0.45
SEID(*α*)	+0.37

**Table 5:** Sensitivity indices for *ℛ*_HP_^0^ = *ℛ*_*P*_^0^.

Sensitivity index	Values
SEID(*β*_2_)	+1
SEID(*μ*_2_)	+0.01
SEID(*γ*)	-0.60
SEID(*ε*_1_)	0.21
SEID(*p*_1_)	-0.72

## Data Availability

Data used to support the findings of this study are included in the article
